# The effect of nutritional risk management program on the growth and development of infants and toddlers with congenital heart disease after discharge

**DOI:** 10.3389/fped.2024.1416778

**Published:** 2024-08-27

**Authors:** Ya-Li Huang, Wen-Yi Luo, Xie-Lei Wang, Feng Zheng, Jian-Hua Gao, Ming-Xia Chen, Yang-Dong Pan

**Affiliations:** ^1^ Department of Cardiac Surgery, Fujian Children’s Hospital (Fujian Branch of Shanghai Children’s Medical Center), Fuzhou, China; ^2^ College of Clinical Medicine for Obstetrics & Gynecology and Pediatrics, Fujian Medical University, Fuzhou, China; ^3^ Shanghai Children’s Medical Center, School of Medicine, Shanghai Jiao Tong University, Shanghai, China

**Keywords:** congenital heart disease, infants and toddlers, nutritional management, nursing care, growth and development

## Abstract

**Objective:**

To evaluate the effect of nutritional risk management program on the growth and development of infants and toddlers with congenital heart disease (CHD) after discharge.

**Methods:**

Infants and toddlers with CHD discharged from a children's specialized hospital in southeast China were selected as the research subjects. The subjects were divided into the intervention group and the control group. The intervention group underwent a nutritional risk management program combined with traditional follow-up after discharge, whereas the control group received traditional follow-up after discharge. The primary outcome measure were the height-for-age Z-score (HAZ), weight-for-age Z-score (WAZ), and weight-for-height Z-score (WHZ) at different time point and the percentage of growth and development curves were also recorded and analyzed.

**Results:**

There were no statistically significant differences in general characteristics between the two groups. However, in the intervention group, the percentages of HAZ < −2, WAZ < −2, and WHZ < −2 were lower than those in the control group at 3rd and 6th months after discharge (*P *< 0.05). The percentage of growth and development curves (3%–97%) was higher than that in the control group (*P* < 0.05). The readmission rate within 6 months after discharge in the intervention group was lower than that in the control group (*P *< 0.05).

**Conclusion:**

Implementing nutritional risk management program for infants and toddlers with CHD after discharge can help improve postoperative malnutrition, promote growth and development and achieve catch-up growth as soon as possible.

## Introduction

Congenital heart disease (CHD) refers to structural abnormalities of the heart and/or macrovascular at birth, which is the most common birth defect (1). Studies have shown that the incidence of acute and chronic malnutrition in children with CHD can be as high as 50%, higher than the average level of hospitalized children during the same period (2, 3). The post-discharge period is a high-risk stage for malnutrition in children with CHD. After surgery, malnutrition persists in 50%–75% of children with CHD, emphasizing the critical importance of early prediction for effective nutritional intervention (4, 5). Simultaneously, children with CHD are in a state of high energy metabolism, and whether the intake of nutrition and calories is sufficient is the key to nutritional improvement.

Medical staff and families should pay attention to the nutritional management of children and comprehensively consider it after discharge. Compared with older children or adults, the growth and development of infants and toddlers are more rapid, and the amount required for growth and development is higher. However, the digestive system function is not yet fully developed, with less energy reserve and poor compensatory ability. If the course of the disease is prolonged or recurrent, it can easily lead to digestive dysfunction, affect the absorption of nutrients. In caring for children with CHD, various strategies need to be integrated to identify and manage malnutrition (6).

There exists a scarcity of research focusing on nutritional risk management for children with CHD post-discharge both domestically and internationally. This study explored the application effects of nutritional risk management programs for infants and toddlers with CHD after discharge, aiming to provide a reference for standardized and scientific nutritional management.

## Material and methods

### Study design

This was a non-concurrent pre-post control study and selected pediatric patients with CHD who were discharged from the cardiovascular center of a children's hospital in southeastern China. Patients with CHD discharged from January 2023 to June 2023 were assigned to the intervention group, while patients discharged from June 2022 to December 2022 were used as control.

### Participant recruitment

Inclusion criteria: (1) simple CHD diagnosed by ultrasonography. (2) Age range from 0 to 3 years old. (3) The parents of the children have basic literacy skills and are able to communicate without obstacles. (4) Parents are willing to participate in this study and have signed the informed consent form. Exclusion criteria: (1) Non-cardiac diseases that lead to nutritional intake disorders, such as gastrointestinal malformations, genetic diseases related to growth restriction, etc. (2) Total parenteral nutrition. (3) The primary caregiver has mental illness or cognitive abnormalities.

### Measurements

Infant Feeding & Nutrition Checklist for Congenital Heart Disease (IFNC:CHD) developed by Canadian scholars Astrid et al. (7) in 2010, is used for feeding and nutritional risk screening for infants and toddlers with CHD. This scale includes three dimensions and 11 items. The three dimensions are rapid screening items (4 items), nutritional assessment items (4 items), and feeding assessment items (3 items). Each item has three answers: “yes”, “uncertain”, and “no”. Answering “yes” to one item indicates having feeding and nutritional risks. This scale has good reliability and validity, the Cronbach's *α* coefficient is 0.804.

The World Health Organization (WHO) endorses the use of Z scores as a standard metric for assessing malnutrition. Z scores for height-for-age (HAZ), weight-for-age (WAZ), and weight-for-height (WHZ) are computed respectively, with the Z value determined by the formula: (observed value - population median)/population standard deviation. According to WHO's growth standards, malnutrition is defined as a Z score < −2. Specifically, WAZ < −2 signifies underweight, reflecting acute malnutrition; HAZ < −2 indicates stunting, representing chronic malnutrition; and WHZ < −2 denotes wasting, which serves as a comprehensive measure of both recent and long-term nutritional status (8).

This study adheres to the WHO-recommended growth and development charts (accessible at http://www.who.int/childgrowth/standards/en/) as the benchmark for evaluating development. Weight, as a vital clinical indicator, reflects physical growth and development. For weight measurement, the infants and toddlers are dressed in a single layer of clothing and placed centrally on a pre-zeroed scale, ensuring no contact with surrounding objects and no movement. Measurements are recorded when the reading stabilizes. For length, a standardized measuring board is utilized, with the infants and toddlers placed supine, head secured against the top of the board, and body aligned straight and close to the board base. To minimize operational error, the same trained individual conducts all weight and height measurements, performing each thrice consecutively and calculating the average for accuracy. According to WHO growth standards, measurements falling within the P3-P97 percentile range are deemed normal, while values below the 3rd percentile (P3) indicate stunting and necessitate prompt intervention.

### Sample size calculation

With the malnutrition (at least one of the three scores, HAZ, WAZ, and WHZ, has a score < −2) as the primary outcome measure for both groups, the patients were followed up for 6 months, and the differences in HAZ, WAZ, and WHZ were calculated, where *P*_1_ = 18% and *P*_2_ = 36% based on the preliminary test results. To compare the difference in the malnutrition between the groups, a two sample independent *t*-test will be employed, setting *α* at 0.05 and ensuring a power of 80%. With a 1:1 ratio between the groups, sample size estimation using PASS 11 indicates that each group will require 74 patients, totaling 148 patients. Considering the potential risk of sample size due to dropout rates, the actual sample size will be 174 patients (148 ÷ 85% = 174), with 87 patients in each group.

### Traditional follow-up after discharge

After discharge, provide routine nutritional guidance, eat small meals multiple times, and gradually increase the feeding amount. Choose high nutrition, high vitamins, and easily digestible foods, avoid overeating and consuming salty foods to avoid increasing the burden on the heart. The WHO recommends that the best feeding method for infants and young children is exclusive breastfeeding from birth to 6 months of age. At the same time, starting from 6 months of age, timely, reasonable, appropriate, and safe addition of complementary foods and nutritional supplements should be provided to meet the nutritional needs of infants and young children. The feeding process follows the “Chinese Infant and Child Feeding Guidelines (2022)” recommended by the Chinese Nutrition Society (9). Routine discharge guidance will be provided including dietary, exercise, medication and other precautions. Outpatient follow-up will be conducted at a frequency of 1 month, 3 months, 6 months, 1 year, and annually thereafter.

### The nutritional management program

**Figure 1:**
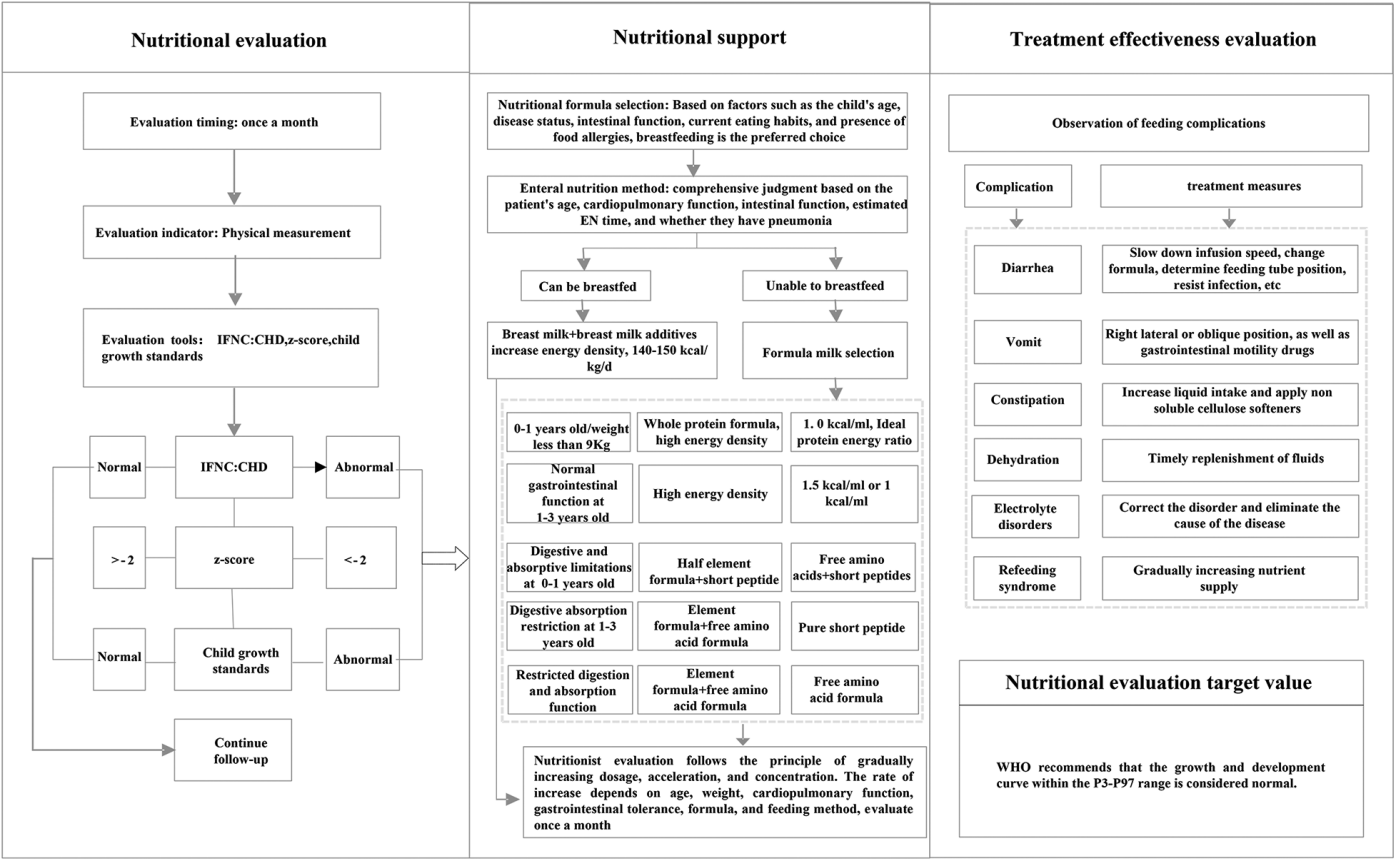
The nutritional risk management process.

The nutritional management program's implementation entailed several key strategies, which can serve as guidance for integrating similar practices into daily clinical routines. (1) Establish a nutrition support team consisting of a cardiologist, a nurse specialist, and a dedicated nutritionist. This team underwent rigorous training, covering nutritional assessment methodologies, the administration of nutritional support, and the evaluation of treatment efficacy. This training ensured that each team member was proficient in the comprehensive management process, fostering seamless collaboration and enhanced patient care. (2) Establish a follow-up information management group. This group was created to facilitate real-time communication and guidance for patient families. Post-discharge, a standardized nutrition management protocol was adhered. If any unusual or abnormal situation occurred, the attending cardiologist was promptly notified, and the nutrition department was consulted for specialized input. Nutrition intervention was then implemented in accordance with the nutritionist's consultation recommendations. Regular follow-ups, including at discharge and monthly post-surgery, ensured timely and personalized nutritional guidance was provided to patients based on their evolving condition. (3) Implement the nutrition risk management program. The real-time registration and updating of patient information to maintain data accuracy and currency. The utilization of regular communication channels, including messaging services, to facilitate timely follow-up visits. Growth and development were tracked diligently, with height, weight, and feeding status recorded meticulously. Monthly summaries were prepared and promptly shared with physicians and nutritionists for review and feedback. The integration of a warning system, which alerted healthcare providers if a child's growth and development failed to improve within a specified timeframe. In such instances, the nurse would remind the family to seek medical attention promptly, facilitating the timely adjustment of the treatment plan. The nutritional risk management process was shown in Figure 1.

### Outcome measures

The primary outcome measure for this study were the incidence of malnutrition (including stunting, underweight and wasting), percentage of growth and development curves. Secondary outcome measures encompassed: morbidity rate within 6 months after discharge (including respiratory infections, feeding intolerance, arrhythmia, liver and kidney dysfunction, etc.) and readmission rate (referring to readmission after discharge).

### Data collection methods

Collect general information and disease-related information including gender, age, height, delivery mode, premature birth, disease type, operation time, cardiopulmonary bypass time, and relationship with caregivers. Collect the height, weight, and feeding status of the control group at discharge, 1st month, 3rd months, and 6th months after discharge. Collect the monthly height, weight, and feeding status of the intervention group at discharge and within the first 6 months after discharge. Record the number of morbidity and readmissions of the two groups within 6 months after discharge.

### Statistical analysis

The SPSS 25.0 software was used for statistical analysis. Continuous data are presented as the mean ± standard deviation (SD). The continuous data conformed to a normal distribution by the normal distribution test. Continuous data between the two groups were compared by t-test, qualitative data between the two groups were compared by Chi square test. Kaplan–Meier analyses was used to compare changes in readmission rate and morbidity rate between the two groups and Log Rank method for pairwise comparison between groups. A p-value of <0.05 was defined as a statistically significant difference.

## Results

### Baseline characteristics and disease homogeneity

A total of 174 participants were included in the study. No signiﬁcant differences were found in gender, age, height, weight, delivery mode, premature birth, family monthly income, operation time, cardiopulmonary bypass time and caregiver relationship between the two groups. There was also no signiﬁcant difference in the types of CHD between the two groups, the Chi square test *p*-value for the types of CHD (including ASD, VSD, PDA, and others) was 0.841. If others category was not included, the *p*-value for ASD, VSD and PDA combined was 0.706 (*P* > 0.05) (Table 1).

**Table 1:** Characteristics of the patients with CHD in two groups.

Variables		Intervention group (*n* = 87)	Control group (*n* = 87)	*t/χ* ^2^	*P*
Gender, *n* (%)	Male	44 (50.575)	45 (51.724)	0.023	0.879
	Female	43 (49.425)	42 (48.276)		
Age (months), mean (SD)		6.299 ± 5.859	7.363 ± 6.207	0.985	0.326
Height (cm), mean (SD)		63.957 ± 10.486	63.466 ± 11.369	0.297	0.767
Weight (Kg), mean (SD)		6.226 ± 2.743	5.985 ± 2.534	0.604	0.546
Delivery mode, *n* (%)	Vaginal delivery	57 (65.517)	58 (66.667)	0.026	0.873
	Cesarean section	30 (34.483)	29 (33.333)		
Premature birth, *n* (%)	Yes	9 (10.345)	13 (14.943)	0.833	0.362
	No	78 (89.655)	74 (85.057)		
Family monthly income (yuan), *n* (%)	<5,000	41 (47.126)	37 (42.529)	0.383	0.826
	5,000∼10,000	39 (44.828)	42 (48.276)		
	>10,000	7 (8.046)	8 (9.195)		
Types of CHD, *n* (%)	ASD	8 (9.195)	11 (12.644)	0.532	0.466
	VSD	52 (59.770)	47 (54.023)	0.586	0.444
	PDA	10 (11.494)	10 (11.494)	0.000	1.000
	others	17 (19.541)	19 (21.839)	0.140	0.708
Operation time (h), mean (SD)		2.742 ± 0.720	2.991 ± 0.953	1.949	0.053
CPB time (h), mean (SD)		1.037 ± 0.597	1.121 ± 0.722	0.833	0.406
Caregiver relationship, *n* (%)	Parents	81 (93.103)	79 (90.805)	0.311	0.577
	Non-parents	6 (6.897)	8 (9.195)		

ASD, atrial septal defect; VSD, ventricular septal defect; PDA, patent ductus arteriosus; others refer to the presence of two or three types among ASD, VSD, and PDA; CPB, cardiopulmonary bypass.

### Post-discharge outcomes and intervention effectiveness

There was no statistically significant difference between the two groups at discharge and the 1st month after discharge in terms of HAZ < −2 (stunting), WAZ < −2 (underweight), and WHZ < −2 (wasting). At the 3rd and 6th months after discharge, HAZ < −2, WAZ < −2, and WHZ < −2 of the intervention group were lower than those in the control group (Table 2). At the 3rd and 6th month after discharge, the percentage of growth and development curves (3%–97%) in the intervention group was higher than that in the control group (Table 3). There was no statistically significant difference in the rate of morbidity within 6 months after discharge between two groups. The readmission rate after discharge within 6 months in the intervention group is lower than that in the control group (Table 4).

**Table 2:** Comparison of the incidence of malnutrition between two groups.

Variables		Intervention group (*n* = 87), *n* (%)	Control group (*n* = 87), *n* (%)	*χ* ^2^	*P*
HAZ<−2	At discharge	33 (37.931)	25 (28.736)	1.655	0.198
	1st month after discharge	20 (22.989)	22 (25.287)	0.126	0.723
	3rd month after discharge	10 (11.494)	21 (24.138)	4.749	0.029
	6th month after discharge	7 (8.046)	16 (18.390)	4.058	0.044
WAZ<−2	At discharge	41 (47.126)	45 (51.724)	0.368	0.544
	1st month after discharge	24 (27.586)	36 (41.379)	3.663	0.056
	3rd month after discharge	19 (21.839)	34 (39.080)	6.105	0.013
	6th month after discharge	5 (5.747)	15 (17.241)	5.649	0.017
WHZ<−2	At discharge	28 (32.184)	24 (27.586)	0.439	0.508
	1st month after discharge	17 (19.540)	20 (22.989)	0.309	0.578
	3rd month after discharge	12 (13.793)	23 (26.437)	4.328	0.037
	6th month after discharge	7 (8.046)	18 (20.690)	5.652	0.017

**Table 3:** Comparison of the percentage of growth and development curves after discharge between two groups.

Variables		Intervention group (*n* = 87), *n* (%)	Control group (*n* = 87), *n* (%)	*χ* ^2^	*P*
At discharge	<3%	25 (28.736)	27 (31.034)	0.110	0.740
	3%∼97%	62 (71.264)	60 (68.966)		
1st month after discharge	<3%	18 (20.690)	20 (22.989)	0.135	0.714
	3%∼97%	69 (79.310)	67 (77.011)		
3rd month after discharge	<3%	10 (11.494)	20 (22.989)	4.028	0.045
	3%∼97%	77 (88.506)	67 (77.011)		
6th month after discharge	<3%	4 (4.598)	12 (13.793)	4.405	0.036
	3%∼97%	83 (95.402)	75(86.207)		

**Table 4:** The survival time of the different groups of patients.

Variables		*n* (%)	Survival time [day, 95% CI]	*χ* ^2^	*P*
Readmission rate	Intervention group (*n* = 87)	3 (3.448)	176.851 (95% CI: 173.220–180.481)	3.956	0.047
	Control group (*n* = 87)	10 (11.494)	172.218 (95% CI: 167.157–177.280)		
Postdischarge morbidity rate	Intervention group (*n* = 87)	2 (2.299)	177.563 (95% CI: 174.195–180.931)	2.845	0.092
	Control group (*n* = 87)	7 (8.046)	173.931 (95% CI: 169.304–178.558)		

## Discussion

While mortality rates for children with CHD have significantly declined, there is a growing population of individuals with CHD living into adulthood prompting the need to optimise long-term development and quality of life (10). Malnutrition remains an important issue for children with CHD after discharge. After surgical treatment, the abnormal anatomical structure of the heart in children with CHD can be corrected, and theoretically, the total resting energy consumption of the body can return to normal. Timely supply of sufficient nutrition after surgery can help improve malnutrition in children, leading to rapid growth improvement (11). Usually, most children follow a certain pattern or track of normal growth and development, while CHD children may deviate from the normal growth pattern due to their disease reasons. The pooled estimates of preoperative malnutrition in children with CHD were 27.4% for underweight, 24.4% for stunting, and 24.8% for wasting. Catch-up growth was found in the postoperative period among some children (12).

The incidence of malnutrition in CHD infants is high, and early nutritional assessment and intervention play an important role in their treatment and improvement of efficacy. Nutritional assessment and management of CHD infants can improve their prognosis (13). With the improvement of surgical techniques and postoperative ICU treatment levels, the trend towards younger patients undergoing CHD surgery is becoming increasingly apparent. The earlier the surgery is performed, the more advantageous it is for children to catch up with their peers in terms of growth and development. However, the earlier the surgery time, the higher the various risks faced by children, including nutritional risk, and the greater the challenge of nutritional support (14). In some studies targeting cyanotic CHD, 15%–50% of patients have less or even no postoperative catch-up growth (15). Therefore, nutritional management of infants and toddlers with CHD after discharge should be taken seriously and comprehensively considered.

The American society for extracorporeal and enteral nutrition and the European society for pediatric gastroenterology, hepatology, and nutrition both recommend conducting nutritional risk screening for children at risk, and conducting nutritional assessments and interventions to improve nutritional status, thereby reducing the incidence of complications, shortening hospital stay, and promoting disease prognosis (16). The study showed that 43% of children with CHD still suffer from malnutrition three months after surgery, and the nutritional status of most children can recover to normal levels of the same age one year after surgery (17). The situation of postoperative malnutrition in children with CHD is severe, with an incidence of malnutrition at discharge of about 25%, higher than the average level of hospitalized children during the same period (18). A meta-analysis on energy metabolism in children with CHD showed that their daily energy expenditure was 376 kJ/kg, a 35% increase compared to normal peers, and their respiratory energy consumption was 3–5 times higher than that of healthy infants (19).

Although surgery has improved heart function to a certain extent, the inhibitory effect of surgery on growth catch-up is still significant. Our study showed that the incidence of malnutrition in the intervention group was lower than that in the control group at the 3rd and 6th months after discharge. The readmission rate of the intervention group was 3.448%, lower than that of the control group. There was no significant difference in the incidence of malnutrition between the two groups at discharge and the 1st month after discharge, which may be due to incomplete recovery of cardiac function after surgery, combined with continuous use of diuretics, which resulted in the patients still being in a state of malnutrition, and their nutritional status did not improve significantly. However, starting from the 3rd months after discharge, the growth and development of infants and toddlers in the intervention group showed significant improvement under nutritional risk management. The nutritional risk management program played a positive role in identifying the risk of malnutrition in infants and toddlers with CHD in the early stage and providing targeted interventions to reduce the readmission rate during follow-up and alleviate the burden on families.

The nutritional risk management program played a role in timely understanding of the nutritional status, closely monitoring and identifying high-risk as soon as possible, and establishing a rapid intervention mechanism to guide clinical physicians in timely, reasonable, and standardized nutritional intervention, reduce the incidence of malnutrition, and promote recovery after discharge. It emphasized the monitoring process of home-based growth and development in infants and toddlers with CHD, including measuring body length and weight, monitoring frequency, evaluation of measurement results, and drawing and evaluating growth curves. This study selected infants and toddlers aged 0–36 months as the research subjects. Compared with older children or adults, the growth and development of infants and toddlers are more rapid and require a higher amount of growth and development. However, their digestive system function is not yet mature, energy reserves are limited, and compensatory ability is poor. If the disease course is long or repeated, it can easily lead to digestive dysfunction, affecting the absorption of nutrients, which is a key focus of medical staff (20).

The results of our study showed that the percentage of growth and development curves in the intervention group (3%–97%) was higher than that in the control group at the 3- and 6-month time points after discharge, which was similar to the research results of Zhang et al. (21), nutrition management strategy for neonates after surgery, remote nutrition management can effectively improve the nutritional status of neonates and promote their growth and development. A diagnosis of CHD warrants regular growth monitoring and assessment of feeding ability. Early referral for nutritional support and speech therapy will improve growth outcomes (22). It appears that the significant improvements in nutrition and growth and development were found at the 3- and 6-month time points, which suggests that feedback after the 1- and 2-month visits led to improved nutritional interventions. Therefore, nutritional risk management program for discharged infants and toddlers with CHD can promote postoperative growth and catch-up growth through standardized nutritional implementation processes and monitoring plans.

This study has some limitations. First, this study was a single-center study. Second, the survey results were compared with the standards of the WHO, without considering ethnic differences. Third, complex CHD populations were not considered and the follow-up time was short. In the future, more centers, large samples, and further verification of the long-term effects of interventions are still needed. In addition, children's nutritional status is closely related to family's economic situation, dietary habits, and psychological and behavioral development, which was not covered in the study. In the future, further exploration of its correlation can be conducted.

## Conclusion

This study implements a nutritional risk management program for discharged infants and toddlers with CHD, which helps to improve postoperative malnutrition, promote growth and development, and achieve catch-up growth as early as possible.

## Data Availability

The original contributions presented in the study are included in the article/Supplementary Material, further inquiries can be directed to the corresponding author.
